# Lifestyle interventions to maternal weight loss after birth: a systematic review

**DOI:** 10.1186/s13643-019-1186-2

**Published:** 2019-12-16

**Authors:** Pernille Kjaergaard Christiansen, Mette Maria Skjøth, Mette Juel Rothmann, Christina Anne Vinter, Ronald Francis Lamont, Eva Draborg

**Affiliations:** 1 Department of Public Health, University of Southern Denmark, Odense, Denmark; 2 Centre for Innovative Medical Technology, Odense University Hospital, Odense, Denmark; 3 Department of Multimedia and IT, University College Lillebaelt, Odense, Denmark; 4 OPEN, University of Southern Denmark, Odense, Denmark; 5 Department of Endocrinology, Odense University Hospital, Odense, Denmark; 6 Steno Diabetes Center Odense, Odense University Hospital, Odense, Denmark; 7 Department of Clinical Research, University of Southern Denmark, Odense, Denmark; 8 Department of Gynecology and Obstetrics, Odense University Hospital, Odense, Denmark; 9 Division of Surgery, University College London, London, UK; 10 Northwick Park Institute of Medical Research Campus, London, UK

**Keywords:** Healthy Life Style, Information and Communication Technology, Postpartum, Intervention, Systematic Review, Weight Control

## Abstract

**Background:**

Over the past decades, there has been an increase in overweight and obesity in women of childbearing age, as well as the general population. Overweight and obesity are related to a later, increased risk of type 2 diabetes and cardiovascular diseases. Increasing weight between pregnancies has a negative impact on the development of the fetus in a subsequent pregnancy. It is also related to long-term obesity and overweight for the woman. Accordingly, weight control in women of the childbearing age is important for both women and their offspring. Information and communication technology (ICT) has become an integrated part of many peoples’ lives, and it has the potential to prevent disease. In this systematic review, we summarize the evidence from randomized controlled trials to compare effects of different ICT-based interventions to support postpartum women to achieve weight loss.

**Methods:**

A systematic search was performed in PubMed, Embase, PsycInfo, CINAHL, Web of Science, Scopus, and Cochrane, searching on terms, such as postpartum, weight loss, telemedicine, and randomized controlled trials. Two independent researchers undertook study selection and data extraction. Results were reported narratively. The systematic review only included studies that were randomized controlled trials.

**Results:**

Eight studies were included in the systematic review. All of them were characterized by applying one or more ICT components to assist postpartum women in weight control, and had weight loss as an outcome measure. A significant difference was found in weight loss between control group and intervention group in the majority of the studies. However, five of the studies had a relatively short follow-up period (40 days to 16 weeks), six of the studies had a relatively small sample size (18 to 66 women), and half of the studies indicated challenges with adherence to the interventions over time.

**Conclusion:**

ICT-based interventions can support postpartum women to achieve a healthy lifestyle and weight control. Future studies should focus on larger sample sizes, longer follow-up periods, and adherence to the interventions.

**Systematic review registration:**

PROSPERO CRD42018080731

## Key message

Maternal obesity is an independent risk factor for adverse maternal and fetal outcomes including gestational diabetes and childhood obesity. ICT-based interventions can support weight loss among postpartum women. Future studies should focus on longer follow-up period, larger sample sizes, and adherence to the interventions.

## Background

Over the past decades, there has been an increase in women of childbearing age as well as in the general population, who are either overweight or obese [1]. The amount of self-reported overweight or obese women in Denmark has increased from 24.5% in 2000 to 27.8% in 2017 and in the USA from 27.9 in 2001 to 28.9% in 2016 [2]. Obesity has a significantly negative impact on fertility, adverse pregnancy outcomes such as gestational diabetes mellitus (GDM) and preeclampsia, and birth outcomes [3]. Obesity compromises the health of the mother and the offspring [4] and is related to increased risk of type 2 diabetes (T2DM) and cardiovascular disease in the mother in later life [5]. 40.9% of women diagnosed with GDM are estimated to develop T2DM within 7 years of their pregnancy [6]. Increasing weight between pregnancies has a negative impact on the development of the fetus in a subsequent pregnancy [7, 8]. It is also related to being obese and overweight long term. Accordingly, weight control among women of childbearing age is important for both women and their offspring.

Several studies indicate that > 50% of all pregnant women with a body mass index (BMI) > 25 kg/m^2^ gain more weight during pregnancy than recommended by the Institute of Medicine (IOM) [9]. The IOM recommends that underweight women should gain between 12.5 and 18 kg during pregnancy, women with a normal BMI should gain between 11.5 and 16 kg, those that are overweight should gain 7 to 11.5 kg, and those that are obese should gain 5 to 9 kg [10]. Gestational weight gain (GWG) exceeding the IOM recommendations is associated with postpartum weight retention [11, 12], which puts the woman at higher risk of developing gestational diabetes mellitus (GDM) in a subsequent pregnancy [4]. In the postpartum period, most women lose some of the weight gained during pregnancy, but in many cases, the women will end up with a higher BMI compared to their pre-pregnancy BMI [7].

Information and Communication Technology (ICT) has become an integral part of many individuals’ everyday lives and has the potential to support prevention of disease and improve lives [13]. ICT can be defined as “ICT refers to technologies that provide access to information through telecommunications. It is similar to Information Technology (IT), but focuses primarily on communication technologies. This includes the Internet, wireless networks, cell phones, and other communication mediums.” [14]. Women of childbearing age use ICT to gain information, including health-related issues during pregnancy, and child development [15]. According to Danish data, 94% of Danish families own a cellphone, and 84% percent own a smartphone [16]. ICT can improve or assist the services provided by the healthcare sector through more customized, efficient, and frequent communication, compared to standard treatments, where communication is face to face or by telephone [17]. Between pregnancies, there is a window of opportunity to focus on weight loss through lifestyle interventions. Starting a family is life changing, and this period can be used to promote a healthy lifestyle and weight control [18, 19]. Recently, there has been an increased interest in the use of ICT to support weight control in relation to pregnancy, and a variety of interventions have been introduced to support weight loss among women in the postpartum period [20]. The objective of this systematic review is to summarize the evidence from randomized controlled trials (RCTs) to compare the effects of different ICT-based interventions to support postpartum women to achieve weight loss.

## Methods

This present study uses a systematic review methodology [21]. The paper is based on the PRISMA statement [22] together with a protocol that has been registered in PROSPERO (CRD42018080731). The synthesis is narrative, as the study designs and samples vary.

### Data sources

A systematic search was performed in PubMed, Embase, PsycInfo, CINAHL, Web of Science, Scopus, and Cochrane. The first author (PKC), assisted by a research librarian, generated a literature search strategy and searched the databases. The literature searches were carried out from May 2017 to February 2018. On the 27th of February 2018, a final search update was made in all databases.

### Search strategy

The search strategy aimed to identify published studies available in full text. A block search strategy, with relevant words, “postpartum,” “weight loss,” “telemedicine,” and “randomized controlled trials” containing both MeSH terms/keywords, and free text, was used together with the terms (AND/OR/NOT). No language or time restrictions were made. RCT filters were applied in PubMed and in Embase, and a filter excluding animal studies was applied in the search in PubMed. The search string is presented in Additional file 1. It was modified for each database. References identified from the search were imported directly into the reference database (EndNote) and later to Covidence. Further publications were sought through the identified articles’ list of references.

#### Inclusion/exclusion criteria for the study selection

The PICO (Population, Intervention [or exposure], Comparison, and Outcome) framework was used to clarify the search [23].

Population: Postpartum women, ≥ 18 years of age participating in an RCT on postpartum weight control. We excluded studies specifically focusing on women with GDM.

Intervention: An ICT-based intervention supporting postpartum women in a healthy lifestyle designed for weight control.

Comparison: The intervention group was compared to a control group that received a standard care in the respective communities, where they live.

Outcome: Weight loss at the end of the intervention period.

**Fig. 1:**
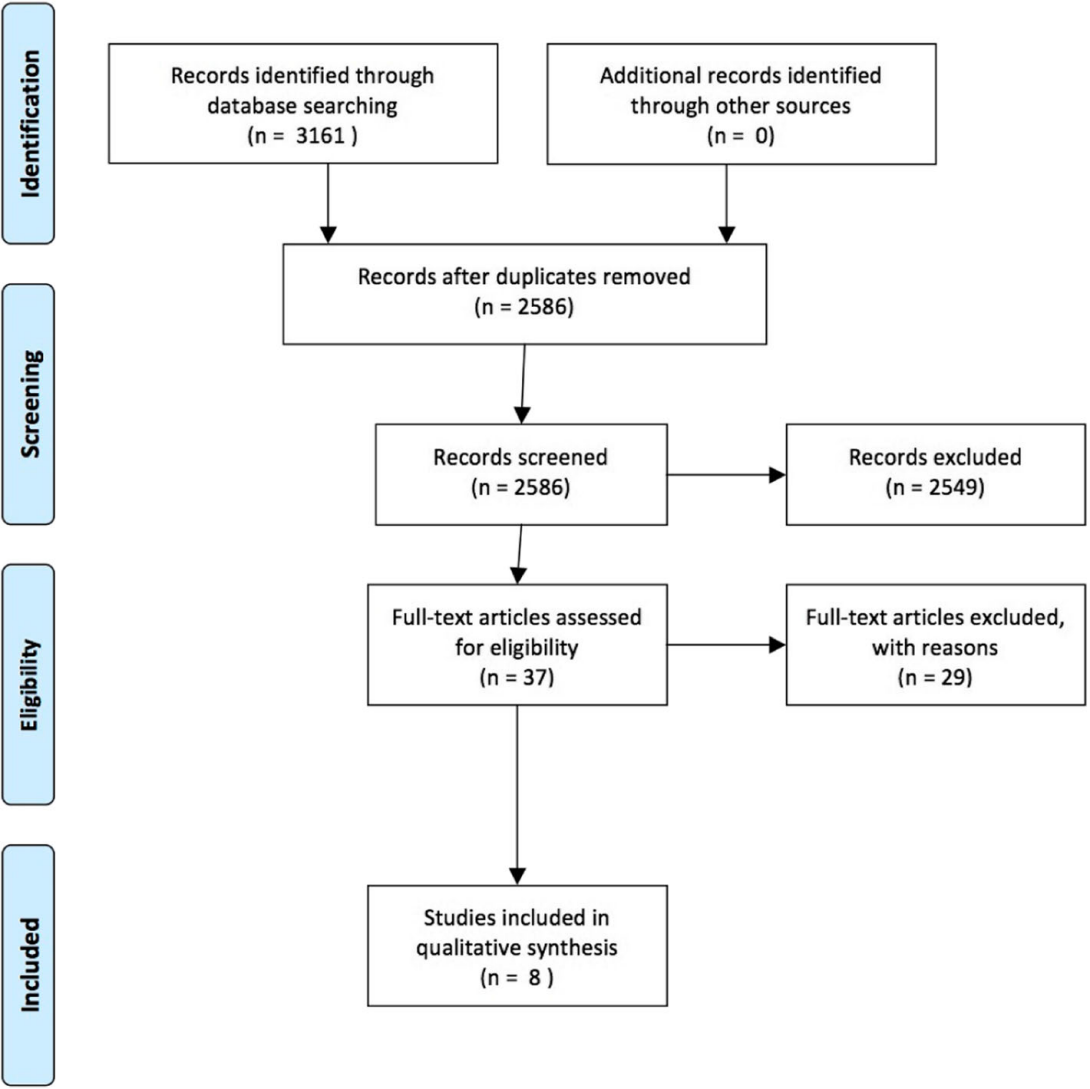
PRISMA flow diagram

Only original publications from peer-reviewed journals about postpartum women and weight loss that were RCTs testing an ICT intervention, and fulfilled the PICO framework, were considered for inclusion in the systematic review. Studies that focused on pregnancy per se were not included. The search, selection, and assessment process were performed in four steps in keeping with the PRISMA flow diagram shown in Fig. 1. The steps were (i) systematic literature search, (ii) removal of duplicates, (iii) identification of potentially relevant articles based on the title and abstract, and (iv) full-text screening. The first author assisted by a librarian performed the search string for the electronic search. Title and abstract were screened in Covidence, which identified a list of duplicates, that were manually checked by the 1st author. A total of 575 articles were removed due to duplicates. First author screened titles and abstracts, and first and second authors read the full text of the remaining articles separately for inclusion or exclusion in the review. Disagreements between the reviewers were resolved by consensus.

### Data extraction and quality assessment

To ensure reliability, and determine the risk of bias in each study, a quality assessment, using Cochrane’s tool for risk of bias in randomized trials, was applied [24]. The checklist comprises seven areas: (i) random sequence generation, (ii) allocation concealment, (iii) blinding of participants and personnel, (iv) blinding of outcome assessment, (v) incomplete outcome data, (vi) selective reporting, and (vii) other bias. As Cochrane’s tool for risk of bias is applied, there are no overall evaluations of each study [24]. An overview of the risk of bias in each study, and scores, is presented in Table 1. The quality of each of the seven criterions were assessed separately and entered in the table with symbols. A “low risk of bias” was estimated, if all or most of the criteria are met (+), “unclear risk of bias” if some of the criteria are met (?), and “high risk of bias” if only few or none of the criteria are met (-). First and second authors reviewed the studies independently accordingly to the quality checklist. Disagreements between the reviewers were resolved by consensus.

**Table 1:** Evaluation of Cochran’s six sources of bias in studies from 2012 to 2017 of weight loss among women who have given birth

	Random Sequence generation	Allocation concealment	Blinding of participants and personnel	Blinding of outcome assessment	Incomplete outcome data	Selective reporting	Other bias
Phelan et al. [19]	(+)	(+)	(+)	(+)	(+)	(+)	(+)
Herring et al. [25]	(+)	(+)	(?)	(?)	(+)	(+)	(+)
Herring et al. [26]	(+)	(-)	(?)	(-)	(+)	(+)	(+)
Gilmore et al. [27]	(+)	(-)	(-)	(-)	(+)	(+)	(+)
Tripette et al. [28]	(+)	(-)	(-)	(-)	(?)	(+)	(+)
Maturi et al. [29]	(+)	(+)	(?)	(-)	(?)	(+)	(+)
Albright et al. [30]	(+)	(+)	(?)	(+)	(+)	(+)	(+)
Colleran and Lovelady [31]	(+)	(+)	(-)	(-)	(-)	(+)	(+)

## Results

The Prisma Flow Chart summarizes the selection of articles found in the systematic review (Fig. 1), and 3161 articles were found through the search in the seven databases. Once duplicates were removed, 2586 articles remained. After screening the title and abstract, based on the criteria from the PICO, 37 articles remained, and after full-text screening, eight studies were left for inclusion in the systematic review [19, 25–31]. Articles excluded in the full-text screening were excluded due to missing ICT in the intervention, or the sample, study design, or outcome did not fulfill the criteria listed in the PICO.

Descriptive information on the included studies is presented in Table 2 at the end of the article and illustrates a list of selected characteristics of each study. The studies were published between 2011 and 2017. Six of the studies were conducted in the USA [19, 25–27, 30, 31], one in Iran [29], and one in Japan [28]. The time span from collecting data to publication varied between 1 year [29] and 4 years [30]. One study did not indicate when data was collected [28]. Although the search was designed to identify postpartum women, one study also included pregnant women but the main focus was on the postpartum period [26]. Baseline characteristics varied between the studies. All studies focused on postpartum women; however, one study did include pregnant women as well [20]. Five studies focused on overweight or obese postpartum women, or women who had exceeded the IOM’s recommendations for weight gain during pregnancy [19, 25–27, 31], while two focused on inactive postpartum women [29, 30], and one study on postpartum women with a BMI 24.9 ± 3.4 kg/m^2^ [28]. Minority groups were also the main focus in some of the American studies (postpartum women on low income) [19, 27] or postpartum women from ethnic minority groups [25, 26]. Seven studies used weight loss (kg) as an outcome [17, 25–29, 31], while one study only examined minutes of moderate to vigorous physical activity (MVPA) [30] as a proxy to weight loss, so the study was included in the systematic review. Five of the studies had a relatively short follow-up period of 40 days to 16 weeks [25, 27–29, 31], while three studies had a follow-up period between 12 and 20 months [19, 26, 30]. A large variation was found between sample sizes of the studies. Six studies had a relatively small sample size, between 18 and 66 participants [26–29, 31], while two studies had larger sample sizes of 311 and 371 participants [19, 30]. Drop out was between 5.5% [25] and 23.73% [19]. Seven of the studies used ICT as a tool to support counseling [19, 25–27, 29–31], while only one study used ICT alone as an intervention [28]. The attrition rate in the eight studies varied from 5.6% [25] to 23.7% [19]. Five studies applied Intention to Treat (ITT) analysis, which indicated a low risk of bias [19, 25–27, 30]. One study used a modified ITT strategy [28], which was unclear with respect to the risk of bias, and two studies did not apply any ITT [29, 31]. Accordingly, their outcome data was unclear. Five of the studies addressed that adherence may decline over time [19, 25–28]. One study divided the intervention group post hoc into low, medium, and high adherence groups, due to a large variance in adherence. Among these, only the group with high adherence (*n* = 5) showed a significant difference in weight loss between the control group and intervention group (− 3.6 to 1.6 kg versus 1.8–0.9 kg; *p* = 0.005) [27]. The post hoc analysis may however have an impact on the power of the sample size, and results should be considered with caution. Finally, one study noted a positive correlation between adherence and weight loss in the intervention group [19]. A financial reward was given for participation in half of the studies [19, 25, 26, 30]. The participants were given between $US 20 per assessment and the opportunity to take part in a raffle of $US 25, when completing a task [25], to $US 120 for full participation [26].

**Table 2:** Study characteristics

Reference	Author/year	Article	Setting	Sample		
[19]	Phelan et al. (2017)	Effect of an Internet-Based Program on Weight Loss for Low-income postpartum women - A Randomized Clinical Trial	California, USA	Postpartum women (6 weeks to 12 months postpartum), BMI above 25, or with a BMI 22–24.9, but exceeding IOM recommendations for GWG with more than 4.5 kg. Age 18–40. English or Spanish speaking, non-smoking, owned a cell-phone, min. 5th grade education.		
[25]	Herring et al. (2014)	Using Technology to Promote Postpartum Weight loss in Urban, Low-Income Mothers: A Pilot Randomized Controlled Trial	Philadelphia, PA, USA.	Postpartum women, who delivered a singleton within the last 2 to 12 months, min. 18 years old, early pregnancy BMI greater than or equal to 25, weight at enrolment that exceeded early pregnancy weight by at least 5 kg.		
[26]	Herring et al. (2017)	Intervening during and after pregnancy to prevent weight retention among African American women	Philadelphia, PA, USA.	African American Women (36% overweight; 64% obese).		
[27]	Gilmore et al. (2017)	Personalized Mobile Health Intervention for Health and Weight Loss in Postpartum Women Receiving Women, Infants, and Children Benefit: A Randomized Controlled Pilot Study	Baton Rouge, Louisiana, USA.	Postpartum women, who gave birth within the past 8 weeks, no younger than 18 years old, overweight or obese (BMI from 25 up to 40), certified for WIC postpartum services, and English speaking.		
[28]	Tripette et al. (2014)	Home-Based Active Video Games to Promote Weight Loss during the Postpartum Period	Tokyo Metropolitan Area, Japan	Postpartum women with a BMI 24.5 ± 3,4. 3 months to 1 year postpartum		
[29]	Maturi et al. (2011)	Effect of physical activity intervention based on a pedometer on physical activity level and anthropometric measures after childbirth: a randomized controlled trial	Abadan, Iran	Postpartum women who had given birth 6 weeks to 12 months prior		
[30]	Albright et al. (2014)	Effectiveness of a 12-month randomized clinical trial to increase physical activity in multi-ethnic postpartum women: Results from Hawaii’s Nā Mikimiki Project	Hawaii, USA	Healthy, postpartum women, who were not regularly active (< 30 min moderate to vigorous physical activity/week); 18–45 years of age; BMIs from 18.5 to 40; infants between 2 and 12 months.		
[31]	Colleran and Lovelady (2012)	Use of MyPyramid Menu Planner for Moms in a Weight-Loss Intervention during Lactation	Minnesota, USA	Mothers between 23 and 37 years of age with full-term (37 weeks) infants less than 3 weeks old, self-reported postpartum body mass index (BMI; calculated as kg/m2) between 25 and 30, fully breastfeeding, 3 days a week of structured physical activity for the past 3 months, cleared by their physician to participate in exercise		
Reference	Data gathering (year)	Follow-up period	Outcome measure(s)	Sample Size	Drop out/percentage	Significant change?
[19]	2011–2015	12 months	Weight loss, physical activity and diet change	*N* = 371	88 persons/23.72%	Yes
[25]	No information	14 weeks	Weight loss, physical activity, and energy intake	*N* = 18	1 person/5.55%	Yes, weight loss and energy intake. No change in physical activity
[26]	2013–2014	16–20 months	Weight loss (percentage of women who regain or are below their early pregnancy weight by 6 months postpartum/12 months postpartum	*N* = 66	10 persons/15.15%	Yes, at 6 months postpartum, but not at 12 months postpartum
[27]	No information	16 weeks	Weight loss, vital signs, circumferences, body composition	*N* = 40	5 persons/12.50%	No, only within high adherence groups (body weight and body fat reduction)
[28]	No information	40 days	Weight loss, improve body composition	*N* = 34	4 persons/11.75%	Yes
[29]	2010	12 weeks	Physical activity, weight loss	*N* = 66	4 persons/6.06%	Yes
[30]	2008–2011	12 months	Moderate-to-vigorous physical activity/week	*N* = 311	62 persons/19.94%	Yes, but only among those with low MVPA at baseline.
[31]	2008–2010	16 weeks	Weight loss, energy intake	*N* = 31	4 persons/12.90%	Yes
Reference	Adherence issues	Published protocol?	Intention to treat (ITT)	ICT component(s)	ICT alone or as a supplement to counseling?	Voucher/present
[19]	Yes, there is a correlation between adherence and results	Yes	Yes	Website, text messages, accelerometer	ICT is a supplement to counseling.	25 dollars for completing baseline and 6 months’ assessment, and 50 dollars for the 12 months’ assessment.
[25]	Yes, over time there is an issue with adherence	Not mentioned	Yes	Healthy 4Baby, Daily text messages, Bi-weekly phone calls from a coach, Training skills through Facebook	ICT is a supplement to counseling.	20 dollars per assessment/raffle 25 dollars for giving feedback
[26]	No problem with adherence. Text messages used to avoid lack of adherence	Not mentioned	Modified ITT approach	Text messages, Being Healthy4Baby, Facebook, Website, Phone calls	ICT is a supplement to counseling.	100% attendance = 120 dollars voucher for time/travel
[27]	Yes	Not mentioned	Yes	Smart Loss Application, SmartPhone, Body trace scale, FitBit Zip accelerometer (Bluetooth), Phone, E-mail, Text messages, Sense wear armband.	ICT is a supplement to counseling.	Not mentioned
[28]	No problem with adherence, but it is discussed that over time it could be an issue	No, but the authors have made one that has been approved	Half fulfilled	Video game, Nintendo Wii, CD, Game, Wii console, email	ICT stands alone.	Not mentioned
[29]	No problem with adherence	Not mentioned	Not fulfilled	Pedometer, Text messages, Phone calls	ICT is a supplement to counseling.	Not mentioned
[30]	Not mentioned	No, but the authors have made one that has been approved	Yes	Phone calls, accelerometer, website	ICT is a supplement to counseling.	60 dollar gift card for participation
[31]	Not mentioned	No, but the authors have made one that has been approved	Not fulfilled	Email account, MyPyramidPlanner,	ICT is a supplement to counseling.	Not mentioned

The present review shows mixed results with respect to blinding. While the authors of one study claimed to follow the guidelines for blinding of participants and personnel [19], four studies only partially fulfilled the criteria [25, 26, 29, 30], and three of the studies did not mention blinding of participants and personnel [27, 28, 31]. When assessing blinding of outcome, the authors of two studies declared that they followed the guidelines [19, 30], one study had some degree of blinding [25], while five studies did not mention blinding of outcome assessment [26–29, 31]. All studies suggested low risk of bias with respect to “random sequence,” “selective reporting,” and “other bias.” Five studies were assessed as having a low risk of bias for “allocation concealment” [19, 25, 29–31], while three studies suggested a high risk of bias [26–28]. Four studies had registered their trial in Clinical.trials.gov [19, 26, 27, 30], and one in ISRCTN. registry [28], while three studies recorded that the trials had been approved by a Review Board [26, 28, 31].

The present study was carried out to identify interventions that were effective with respect to weight loss, with the purpose of using these results in a further analysis. The interventions can be divided into categories according to their primary ICT component which were (1) Internet-based interventions, (2) text messages, (3) pedometer/accelerometer, (4) gaming, and (5) mobile application. Table 3 gives an overview of the categories in each intervention. The categories are described in detail below.

1. Internet-based interventions

**Table 3:** Overview of ICT-based categories in the eight interventions

Reference	Internet based intervention	Text messages (SMS [short message system])	Pedometer/accelerometer	Gaming	Mobile application
Phelan et al. [19]	X	X	X		
Herring et al. [25]		X	X		
Herring et al. [26]		X	X		
Gilmore et al. [27]		X	X		X
Tripette et al. [28]				X	
Maturi et al. [29]		X	X		
Albright et al. [30]	X		X		
Colleran and Lovelady [31]	X				

MyPyramid Menu Planner [31], Fit Moms/Mamas Activas [19], and The Na Mikimiki intervention [30] examined the effect of an Internet-based intervention. Participants in MyPyramid Menu Planner [31] were given a customized account, where each participant entered dietary intake and physical activity three times each week. Both the participant and a dietician could access the account. Face-to-face counseling was used to introduce the account, and for counseling each week during the intervention period. In Fit Moms/Mamas Activas [19], participants received computerized feedback, text messages, face-to-face group meetings once a month at a clinic, and a weight and physical tracker. The participants also received access to a website, a web diary, and instructional videos. Participants in The Na Mikimiki intervention [30] were given access to a website and telephone counseling, where motivational techniques to overcome barriers and set future goals were used. The website contained tailored information about physical activity and newsletters. The intervention was based on Resnicow’s framework for creating culturally sensitive interventions [32].

MyPyramid Planner and Fit Moms/Mamas Activas both showed a significant change in weight loss between the intervention group and the control group. In My Pyramid Planner, the difference in weight loss was 4.2 kg (*p* < 0.03) [31]. In Fit Moms/Mamas Activas, the difference was 2.1 kg at 6 months (*p* < 0.001) and 2.3 kg at 12 months (*p* < 0.001) [19].

The Na Mikimiki intervention [30] used MVPA as a proxy to weight loss. This study also showed a significant difference in self-reported MVPA, between the intervention group and the control group, with an average of 44 to 246 min/week versus 46 to 146 min/week respectively (*p* = 0.027). Women with two or more children had a significantly increased MVPA compared to women with only one child, and the increase was greater in the intervention group compared to the control group (*p* = 0.016). Participants with low MVPA below the medium at baseline demonstrated a greater increase in MVPA compared to those with MVPA above the medium (*p* = 0.053), but without statistically significance at the 5% level.

2) Text messages (SMS [short message system])

All eight interventions included in the systematic review contained phone calls and/or text messages and/or emails as reminders, to give feedback, information, motivation, counseling, or to allow participants to ask questions. Most of the text messages and phone calls were tailored to the need of each participant. Text messages were the main component in two of the interventions: “The Healthy4Baby” and “Being Healthy for Baby” [25, 26], both of which are based on an intervention with daily text messages tailored to behavioral goals and reminders about returning information on calorie intake and physical activity. Facebook posts with video and links to websites were sent to participants, and each participant in the intervention group received weekly or monthly phone calls by a health coach, a digital scale for self-weighing, and a pedometer. Only the Healthy4Baby intervention showed a significant difference in weight loss between the intervention group and the control group after 14 weeks, with a weight difference equivalent to (3.2 kg; *p* = 0.04), while Being Healthy for Baby intervention only showed a significant difference in the number of participants who were at, or below, their early pregnancy weights at 6 months postpartum (56% versus 29%; *p* = 0.04), but not 12 months postpartum (41% versus 38%; *p* = 0.83).

3) Pedometer/accelerometer

The study by Maturi et al. is a physical activity intervention, based on a pedometer recording daily steps [29]. A counseling session at baseline, weekly reminders by text message, a phone call every second week, and a pamphlet after 8 weeks supported the intervention. After 12 weeks, a significant difference between the intervention group and the control group was found in physical activity and energy expenditure per week (4394 versus 1651 calories; *p* < 0.001) together with a significant difference in weight loss (66.8 kg pre- and 64.7 kg post-intervention; *p* = 0.001) compared to the control group (63.9 kg pre- and 63.9 kg post-control; *p* = 0.001). Five of the other studies included in the systematic review equipped the participants with an accelerometer or pedometer to measure physical activity as a part of the intervention (during the intervention period) [19, 25–27, 30].

4) Gaming

The Active Video Games (AVG) intervention [28] was based on the Nintendo Wii game. Each participant in the AVG group was provided with the game, a Wii console, and all necessary equipment. Participants were recommended to play the AVG for 30 min each day during the intervention period. No further recommendations were given. A significant difference in weight loss between the intervention group and the control group was found after 40 days (− 2.2 ± 0.9 kg versus − 5 ± 0.7 kg, *p* < 0.001). However, three of the fourteen participants in the intervention group complained about injuries from the use of the game. The follow-up period for the Nintendo Wii was only 40 days. None of the other interventions employed gaming elements in their interventions.

5) Mobile application

The SmartLoss application was given to the intervention group called “E-Moms,” which is one form of individualized intervention with continuous surveillance [27]. The intervention consisted of a mobile application and a clinician dashboard, which made it possible for the clinician to monitor all E-Moms simultaneously, and potentially increase adherence to the intervention, and customize counseling. Based on weight development, each participant received instructions (SmartTips) on how to adjust calorie intake and set new goals for behavioral changes. The intervention also included an accelerometer, and a fictitious new “mom” called “Mia” who was going through a weight loss journey. A significant difference in weight loss was only present among the high adherence users in the intervention group (− 3.6 ± 1.6 kg versus 1.8 ± 0.9 kg; *p* = 0.005). However, this group consisted of only five participants.

## Discussion

The overall results show that ICT-based interventions, to some degree, support weight loss among postpartum women, which is also concluded by an existing review on e-health interventions for pregnant and postpartum women [20]. The studies included were pragmatic and were aimed to represent daily practice, compared to explanatory studies [33]. Pragmatic studies are at risk of being influenced by external conditions, as they are less controlled compared to explanatory studies. Individuals who volunteer to participate in a pragmatic study are often healthier, and/or better educated, and consequently some of the potential users may not be present in the studies, which may bias the generalizability.

The use of a narrative methodology contains limitations with respect to statistical analysis and has been criticized for being too narrow [34]. However, the methodology contains the strength that it can demonstrate not only what works, but also why and how it works. Heterogeneity between the studies is a limitation, as there is a wide variation between the included studies, with respect to the intervention, the setting, population, sample size, adherence to the intervention, follow-up period, blinding of participants and personnel, and blinding of outcome assessment. In addition, what is defined as standard care provided to both the intervention and control groups varies between studies. The differences make it difficult to compare the studies directly. However, the results demonstrate possible approaches to weight loss among postpartum women. Five of the studies had a relatively short follow-up period, which made the results uncertain, as participants, over time, may have lost interest in the intervention, which lowers the effect of the intervention. One of the studies with a long follow-up period only showed a significant change after 6 months, but not at 12 months [26]. In addition, the majority of the studies used a small sample size of 18 to 66 participants, which can be associated with increased uncertainty of the measured effect.

The contexts, in which the interventions were tested, may have had an impact on the results and limited external validity, as both the setting and the characteristics of the individuals in the samples may vary between countries. Statistical health data from the Organisation for Economic Co-operation and Development (OECD) also indicated a difference in lifestyle with respect to food consumption between countries [35]. Some of the studies included considered minority groups rather than the general population. However, a particular intervention targeted at a limited population may be beneficial to this specific group of people. Six of the studies were conducted in the USA, and thus, the overall results are found in a US context, but these do not necessarily present an image of how it works in the rest of the world. However, they demonstrate what can work in a US setting. All of the studies conducted in the USA used ICT as a supplement to counseling, and all had some kind of interaction between care provider or coach and the postpartum women. In addition, most of these studies did show a significant change, though mainly in the short run and among those with a high adherence to the interventions.

The financial reward can be seen as an extrinsic motivation for participants to take part in the study and so may improve adherence. The costs of providing a financial reward should therefore be evaluated as a part of the intervention. With the exception of The Nintendo Wii intervention [28], all the included studies contained a coach or healthcare provider to assist the participants by phone, text message, and/or face-to-face meetings. The intervention MyPyramidPlanner had a research assistant to travel to the women’s homes up to three times a week to assist with childcare, facilitate the exercises, and have a weekly face-to-face talk about dietary intake [31]. Accordingly, the ICT-part of the interventions can be seen as a supplement to personal counseling, and personalized counseling is important for achieving weight loss goals [36]. However, the Nintendo Wii also showed a significant change in weight loss, so ICT interventions without counseling may be successful by using gaming.

Seven of the studies are unclear about blinding or the extent of blinding in the study and implies a risk of bias [25–31]. However, it has to be recognized that blinding is not always possible, as it is conceivable that participants know they are part of a study, and thus the validity of the results are weakened. The present study focuses solely on RCTs, so we have excluded results from studies using other methods.

Previous systematic reviews suggest that IT interventions can support weight reduction among postpartum women and suggest that the successful components of an intervention are personalized communication and frequent interaction with the intervention [20, 37]. The present study indicates that a financial reward or gaming elements are also useful components, though further research on this area is required. The present study also includes additional studies compared to those included in the previous systematic reviews. Future studies should use longer follow-up periods, larger sample sizes, and methods to increase adherence to the interventions through counseling, feedback, rewards, and potentially also gaming elements [37].

## Conclusion

The results from this study show that ICT-based interventions can assist weight loss. The main features of studies that demonstrated a significant change in weight contained elements of feedback, personal coaching, and frequent interaction with the intervention, gaming elements, or a financial reward. Significant change in weight loss appeals to motivation through the fun of gaming and may improve adherence (but replication is needed in more studies). Hence, future trials should take these features into account. It must be taken into account that only two of the studies had more than 100 observations, and only three studies had follow-up periods that were equal to or over a year. Thus, in the planning of future studies, one should carefully consider larger sample sizes, and longer follow-up periods.

## Supplementary information

**Additional file 1.** Search string.

## Data Availability

Not applicable

## Abbreviations

GDM: Gestational Diabetes Mellitus
GWG: Gestational Weight Gain
ICT: Information and communication technology
IOM: Institute of Medicine
OECD: Organisation for Economic Co-operation and Development
RCT: Randomized Controlled Trial
T2DM: Type 2 diabetes mellitus
