# Supplementary material for: Lifestyle interventions to maternal weight loss after birth: a systematic review
Source: Syst Rev. 2019 Dec 16;8:327. doi: 10.1186/s13643-019-1186-2 (PMC6912999; doi:10.1186/s13643-019-1186-2)
Supplement: Supplementary file 1 — Additional file 1. Search string. [file 13643_2019_1186_MOESM1_ESM.docx]

**PubMed**

(((((("Computer Communication Networks"[Mesh]) OR "Telemedicine"[Mesh]) OR ((((((((((social media[MeSH Terms]) OR ((("Multimedia"[Mesh]) OR "Internet"[Mesh]) OR "Therapy, Computer-Assisted"[Mesh]) OR "Computers"[Mesh]) OR) OR "Health Information Systems"[Mesh]) OR "Online Systems"[Mesh]) OR ( "Cell Phones"[Mesh] OR "Smartphone"[Mesh] )) OR "Electronic Mail"[Mesh]))))) OR ((Multimedia OR internet OR tele monitoring OR computer based OR computer assisted OR computer OR web-based OR patient portal OR online OR on-line OR telecommunication OR tele-communication OR webpage OR eHealth OR telehealth OR homepage OR home-page OR website OR world wide web OR www OR phone* OR mobile technology OR communication network OR remote consultation OR electronic health OR ehealth OR mobile health OR m-health OR Internet OR SMS OR text message* OR electronic mail OR e-mail OR social media OR Facebook OR FB OR Skype OR device OR Health Information Systems OR cell phone OR smart phone OR telehealth OR mobile phone OR Medical information systems OR Cellular phone OR Telemedicine OR tele medicine OR tele-medicine OR telemedical OR tele-medical OR telehealth OR tele-health OR telecare OR tele care OR tele-care OR ehealth OR e-health OR remote care OR remote caring OR remote communication OR telecommunication OR tele-communication OR telecommunications OR tele-communications OR Teleconsultation OR Teleconsultations OR App OR Apps OR Health app OR Health apps OR Medical informatics applications OR Software OR Handheld computer OR Handheld computers OR chat OR chat room* OR communication platform* OR blog* OR tailored OR Customized OR Individualized OR self-monitoring OR online consultation* OR online group* OR Bluetooth)))))))

AND

((((((((((((((("Clinical Trials as Topic"[Mesh] OR "Double-Blind Method"[Mesh] OR "Single-Blind Method"[Mesh] OR "drug therapy"[Subheading] OR "Research Design"[Mesh] OR "Placebos"[Mesh] OR "Random Allocation"[Mesh] OR "Randomized Controlled Trials as Topic"[Mesh] OR "Double-Blind Method"[Mesh] OR "Randomized Controlled Trial" [Publication Type] OR "Practice Guideline"[Publication Type] OR "Clinical Trial"[Publication Type] OR "Controlled Clinical Trial"[Publication Type] OR single blind[Title/Abstract] OR single blinded[Title/Abstract] OR single masked[Title/Abstract] OR double blind[Title/Abstract] OR double blinded[Title/Abstract] OR double masked[Title/Abstract] OR triple blind[Title/Abstract] OR triple blinded[Title/Abstract] OR triple masked[Title/Abstract] OR double-blind* OR random allocation[Title/Abstract] OR random allocations[Title/Abstract] OR random allocated[Title/Abstract] OR randomly allocated[Title/Abstract] OR clinical trial[Title/Abstract] OR clinical trials[Title/Abstract] OR placebo*[Title/Abstract] OR random* OR trial*[Title/Abstract]) NOT (case report[Title/Abstract] OR letter[Publication Type] OR historical article[Publication Type] OR "review of reported cases" OR multicase review[Publication Type])))))))))))))))))))

AND

(((postpartum OR post-partum OR postnatal OR post-natal OR after birth OR after delivery OR after pregnancy OR postpartum care OR post-partum care OR post-natal care OR postnatal care OR Postpartum period OR post-partum period OR Puerperium)) OR ((((("Postpartum Period"[Mesh]) OR "Postnatal Care"[Mesh]))))))))

AND

(((((("Body Weight Changes"[Mesh]) OR "Obesity"[Mesh]) OR "Overweight"[Mesh]) OR "Weight Reduction Programs"[Mesh])) OR ((overweight OR over-weight OR obese OR obesity OR obesitas OR Adiposity OR Adipose OR Adipositas OR weight gain OR nutritional obesity OR Overnutrition OR over-nutrition OR Body mass index OR BMI OR weight retention OR weight reduction program*)))

NOT ((animals [mh] NOT humans [mh]))

**EMBASE**

exp telemedicine/ or exp telecommunication/

exp interpersonal communication/ or exp social media/

exp Internet/

exp computer/ or exp computer assisted therapy/

exp technology/

exp medical information system/

| exp online system/ |  |
| --- | --- |

exp text messaging/

exp social media/

exp multimedia/

Multimedia or internet or tele monitoring or computer based or computer assisted or computer or web-based or patient portal or online or on-line or telecommunication or tele-communication or webpage or eHealth or telehealth or homepage or home-page or website or world wide web or www or phone* or mobile technology or communication network or remote consultation or electronic health or ehealth or mobile health or m-health or Internet or SMS or text message* or electronic mail or e-mail or social media or Facebook or FB or Skype or device or Health Information Systems or cell phone or smart phone or telehealth or mobile phone or Medical information systems or Cellular phone or Telemedicine or tele medicine or tele-medicine or telemedical or tele-medical or telehealth or tele-health or telecare or tele care or tele-care or ehealth or e-health or remote care or remote caring or remote communication or telecommunication or tele-communication or telecommunications or tele-communications or Teleconsultation or Teleconsultations or App or Apps or Health app or Health apps or Medical informatics applications or Software or Handheld computer or Handheld computers or chat or chat room* or communication platform* or blog* or tailored or Customized or Individualized or self-monitoring or online consultation* or online group* or Bluetooth).mp. [mp=title, abstract, heading word, drug trade name, original title, device manufacturer, drug manufacturer, device trade name, keyword, floating subheading word]

AND

exp puerperium/

exp postnatal care/

(postpartum or post-partum or postnatal or post-natal or after birth or after delivery or after pregnancy or postpartum care or post-partum care or post-natal care or postnatal care or Postpartum period or post-partum period or Puerperium).mp. [mp=title, abstract, heading word, drug trade name, original title, device manufacturer, drug manufacturer, device trade name, keyword, floating subheading word]

AND
exp body mass/ or exp obesity/

exp overnutrition/

exp weight loss program/

exp weight change/ or exp weight gain/

(overweight or over-weight or obese or obesity or obesitas or Adiposity or Adipose or Adipositas or weight gain or nutritional obesity or Overnutrition or over-nutrition or Body mass index or BMI or weight retention or weight reduction program).mp. [mp=title, abstract, heading word, drug trade name, original title, device manufacturer, drug manufacturer, device trade name, keyword, floating subheading word]

**Scopus / Web of Science**

Multimedia OR internet OR “tele monitoring” OR “computer based” OR “computer assisted” OR computer OR web-based OR “patient portal” OR online OR on-line OR telecommunication OR tele-communication OR webpage OR eHealth OR telehealth OR homepage OR home-page OR website OR “world wide web” OR www OR phone* OR “mobile technology” OR “communication network” OR “remote consultation” OR “electronic health” OR ehealth OR “mobile health” OR m-health OR Internet OR SMS OR “text message*” OR “electronic mail” OR e-mail OR “social media” OR Facebook OR FB OR Skype OR device OR “Health Information System*” OR “cell phone*” OR “smart phone*” OR telehealth OR “mobile phone*” OR “Medical information system*” OR “Cellular phone*” OR Telemedicine OR “tele medicine” OR tele-medicine OR telemedical OR tele-medical OR telehealth OR tele-health OR telecare OR “tele care” OR tele-care OR ehealth OR e-health OR “remote care” OR “remote caring” OR “remote communication” OR telecommunication OR tele-communication OR telecommunications OR tele-communications OR Teleconsultation OR Teleconsultations OR App OR Apps OR Health app OR “Health apps” OR “Medical informatics application*” OR Software OR “Handheld computer” OR “Handheld computers” OR chat OR “chat room*” OR “communication platform*” OR blog* OR tailored OR Customized OR Individualized OR self-monitoring OR “online consultation*” OR “online group*” OR Bluetooth

AND

postpartum OR post-partum OR postnatal OR post-natal OR “after birth” OR “after delivery” OR after pregnancy OR “postpartum care” OR “post-partum care” OR “post-natal care” OR “postnatal care” OR “Postpartum period” OR “post-partum period” OR Puerperium

AND

overweight OR over-weight OR obese OR obesity OR obesitas OR Adiposity OR Adipose OR Adipositas OR “weight gain” OR “nutritional obesity” OR Overnutrition OR over-nutrition OR “Body mass index” OR BMI OR “weight retention” OR “weight reduction program*”

**PsykINFO**

Keywords:

exp Social Media/ or exp Social Networks/ or exp Computers/ or exp Technology/ or exp Internet/ or exp Computer Mediated Communication/

| exp TELEMEDICINE/ |  |
| --- | --- |

exp MULTIMEDIA/

exp Computer Assisted Therapy/

exp Information Systems/

exp Computer Assisted Instruction/

exp ONLINE COMMUNITY/ or exp ONLINE THERAPY/ or exp ONLINE SOCIAL NETWORKS/

exp Cellular Phones/

Multimedia OR internet OR tele monitoring OR computer based OR computer assisted OR computer OR web-based OR patient portal OR online OR on-line OR telecommunication OR tele-communication OR webpage OR eHealth OR telehealth OR homepage OR home-page OR website OR world wide web OR www OR phone* OR mobile technology OR communication network OR remote consultation OR electronic health OR ehealth OR mobile health OR m-health OR Internet OR SMS OR text message* OR electronic mail OR e-mail OR social media OR Facebook OR FB OR Skype OR device OR Health Information Systems OR cell phone OR smart phone OR telehealth OR mobile phone OR Medical information systems OR Cellular phone OR Telemedicine OR tele medicine OR tele-medicine OR telemedical OR tele-medical OR telehealth OR tele-health OR telecare OR tele care OR tele-care OR ehealth OR e-health OR remote care OR remote caring OR remote communication OR telecommunication OR tele-communication OR telecommunications OR tele-communications OR Teleconsultation OR Teleconsultations OR App OR Apps OR Health app OR Health apps OR Medical informatics applications OR Software OR Handheld computer OR Handheld computers OR chat OR chat room* OR communication platform* OR blog* OR tailored OR Customized OR Individualized OR self-monitoring OR online consultation* OR online group* OR Bluetooth

AND

Keywords:

exp Postnatal Period/

postpartum OR post-partum OR postnatal OR post-natal OR after birth OR after delivery OR after pregnancy OR postpartum care OR post-partum care OR post-natal care OR postnatal care OR Postpartum period OR post-partum period OR Puerperium

AND

Keywords:
exp Obesity/

exp OVERWEIGHT/

exp Body Weight/ or exp Weight Control/

exp Body Mass Index/ or exp Weight Gain/

overweight OR over-weight OR obese OR obesity OR obesitas OR Adiposity OR Adipose OR Adipositas OR weight gain OR nutritional obesity OR Overnutrition OR over-nutrition OR Body mass index OR BMI OR weight retention OR weight reduction programmes

**COCHRANE**

MeSH descriptor: [Electronic Mail] explode all trees

MeSH descriptor: [Smartphone] explode all trees

MeSH descriptor: [Cell Phones] explode all trees

MeSH descriptor: [Online Systems] explode all trees

MeSH descriptor: [Health Information Systems] explode all trees

MeSH descriptor: [Therapy, Computer-Assisted] explode all trees

MeSH descriptor: [Internet] explode all trees

MeSH descriptor: [Multimedia] explode all trees

MeSH descriptor: [Social Media] explode all trees

MeSH descriptor: [Telemedicine] explode all trees

MeSH descriptor: [Computer Communication Networks] explode all trees

Multimedia or internet or tele monitoring or computer based or computer assisted or computer or web-based or patient portal or online or on-line or telecommunication or tele-communication or webpage or eHealth or telehealth or homepage or home-page or website or world wide web or www or phone* or mobile technology or communication network or remote consultation or electronic health or ehealth or mobile health or m-health or Internet or SMS or text message* or electronic mail or e-mail or social media or Facebook or FB or Skype or device or Health Information Systems or cell phone or smart phone or telehealth or mobile phone or Medical information systems or Cellular phone or Telemedicine or tele medicine or tele-medicine or telemedical or tele-medical or telehealth or tele-health or telecare or tele care or tele-care or ehealth or e-health or remote care or remote caring or remote communication or telecommunication or tele-communication or telecommunications or tele-communications or Teleconsultation or Teleconsultations or App or Apps or Health app or Health apps or Medical informatics applications or Software or Handheld computer or Handheld computers or chat or chat room* or communication platform* or blog* or tailored or Customized or Individualized or self-monitoring or online consultation* or online group* or Bluetooth:ti,ab,kw (Word variations have been searched)

AND

MeSH

MeSH descriptor: [Body Weight Changes] explode all trees

MeSH descriptor: [Body Mass Index] explode all trees

MeSH descriptor: [Weight Reduction Programs] explode all trees

MeSH descriptor: [Weight Reduction Programs] explode all trees

MeSH descriptor: [Overnutrition] explode all trees

MeSH descriptor: [Overweight] explode all trees

MeSH descriptor: [Obesity] explode all trees

overweight or over-weight or obese or obesity or obesitas or Adiposity or Adipose or Adipositas or weight gain or nutritional obesity or Overnutrition or over-nutrition or Body mass index or BMI or weight retention or weight reduction program:ti,ab,kw (Word variations have been searched)

AND

MeSH

MeSH descriptor: [Postnatal Care] explode all trees

MeSH descriptor: [Postpartum Period] explode all trees

postpartum or post-partum or postnatal or post-natal or after birth or after delivery or after pregnancy or postpartum care or post-partum care or post-natal care or postnatal care or Postpartum period or post-partum period or Puerperium:ti,ab,kw (Word variations have been searched)

**CINAHL**

Multimedia OR internet OR tele monitoring OR computer based OR computer assisted OR computer OR web-based OR patient portal OR online OR on-line OR telecommunication OR tele-communication OR webpage OR eHealth OR telehealth OR homepage OR home-page OR website OR world wide web OR www OR phone* OR mobile technology OR communication network OR remote consultation OR electronic health OR ehealth OR mobile health OR m-health OR Internet OR SMS OR text message* OR electronic mail OR e-mail OR social media OR Facebook OR FB OR Skype OR device OR Health Information Systems OR cell phone OR smart phone OR telehealth OR mobile phone OR Medical information systems OR Cellular phone OR Telemedicine OR tele medicine OR tele-medicine OR telemedical OR tele-medical OR telehealth OR tele-health OR telecare OR tele care OR tele-care OR ehealth OR e-health OR remote care OR remote caring OR remote communication OR telecommunication OR tele-communication OR telecommunications OR tele-communications OR Teleconsultation OR Teleconsultations OR App OR Apps OR Health app OR Health apps OR Medical informatics applications OR Software OR Handheld computer OR Handheld computers OR chat OR chat room* OR communication platform* OR blog* OR tailored OR Customized OR Individualized OR self-monitoring OR online consultation* OR online group* OR Bluetooth

AND

postpartum OR post-partum OR postnatal OR post-natal OR after birth OR after delivery OR after pregnancy OR postpartum care OR post-partum care OR post-natal care OR postnatal care OR Postpartum period OR post-partum period OR Puerperium

AND

overweight OR over-weight OR obese OR obesity OR obesitas OR Adiposity OR Adipose OR Adipositas OR weight gain OR nutritional obesity OR Overnutrition OR over-nutrition OR Body mass index OR BMI OR weight retention OR weight reduction program*)))
